# An Interprofessional Approach to Developing Family Psychosocial Support Programs in a Pediatric Oncology Healthcare Setting

**DOI:** 10.3390/cancers17081342

**Published:** 2025-04-16

**Authors:** Erin Turner, Erica H. Sirrine, Valerie McLaughlin Crabtree, D. Andrew Elliott, Ashley Carr, Paula Elsener, Kendra R. Parris

**Affiliations:** 1 Department of Social Work, St. Jude Children’s Research Hospital, Memphis, TN 38105, USA; erin.turner@stjude.org; 2 Department of Psychology & Biobehavioral Sciences, St. Jude Children’s Research Hospital, Memphis, TN 38105, USA; valerie.crabtree@stjude.org (V.M.C.); kendra.parris@stjude.org (K.R.P.); 3 Department of Pediatric Medicine, St. Jude Children’s Research Hospital, Memphis, TN 38105, USA; darrell.elliott@stjude.org; 4 Department of Child Life, St. Jude Children’s Research Hospital, Memphis, TN 38105, USA; ashley.carr@stjude.org; 5 Department of Family, Guest, and Volunteer Services, St. Jude Children’s Research Hospital, Memphis, TN 38105, USA; paula.elsener@stjude.org

**Keywords:** psychosocial standards, pediatric oncology, preventative health model, psychosocial support programs, scope of practice, psychosocial oncology

## Abstract

Background: The Standards for the Psychosocial Care of Children with Cancer and their Families provide a framework for the delivery of psychosocial care to families experiencing pediatric cancer. Similarly, the Pediatric Psychosocial Preventative Health Model (PPPHM) guides intervention approaches by identifying three tiers of psychosocial support based on a family’s level of risk. Employing both the Standards and the PPPHM, we developed a comprehensive three-tiered approach to support the psychosocial needs of families in a pediatric oncology setting. Methods: After publication of the Standards, our institution merged existing psychosocial disciplines into one unified Psychosocial Services department. The new department worked to clearly define the role and scope of each discipline’s practice to ensure the psychosocial needs of patients and families were being comprehensively met. Interprofessional workgroups were established to evaluate and enhance the psychosocial services offered to patients, siblings, and caregivers using a three-tiered model of support. Membership included representation from patients and parent/caregiver advisors to ensure their perspectives were included in program development. Results: Over ten new programs have been developed to enhance the support of families facing pediatric cancer. At the Universal tier, new programs available to all families include caregiver and sibling support groups, a caregiver podcast, and a relationship health toolkit and workshop. At the Targeted tier, psychosocial interventions and parent mentor supports were implemented. At the Clinical/Intervention tier, a partnership was developed with an external tele-mental health company to provide mental health services to caregivers with significant needs or preexisting mental health disorders. Conclusions: Given the complex needs of families facing pediatric cancer, use of an interdisciplinary approach is paramount to successful support throughout the treatment trajectory. By leveraging the expertise and strengths of diverse disciplines with the perspectives of patients and families, new psychosocial programs can comprehensively address the unique challenges of patients and families impacted by illness.

## Simple Summary

When a child is diagnosed with cancer, the entire family is impacted. Depending on the diagnosis and course of treatment, a variety of psychosocial stressors may be present, including but not limited to learning to care for a medically complex child, separating from other family members, loss of income or employment, or internal family conflict. Effective treatment includes psychosocial support for the entire family. Using both the Standards for the Psychosocial Care of Children with Cancer and their Families and the Pediatric Psychosocial Preventative Health Model (PPPHM), we created a unified Psychosocial Services department and Scope of Practice Grid to ensure interventions were developed by each discipline to comprehensively meet the needs of our families. Interventions were established across the framework of a three-tiered model of support based on the family’s level of need. This was guided by the development of a multi-disciplinary task force that includes parent advisors and staff.

## 1. Introduction

The psychosocial needs of children with cancer and their families are extensive [1,2,3,4]. Addressing these needs requires a multidisciplinary and systematic approach. Our institution has used the Pediatric Psychosocial Preventative Health Model (PPPHM) [5] and the Standards for Psychosocial Care of Children with Cancer and their Families [6] to guide us in the development and implementation of psychosocial support initiatives that will best support our patients and their families.

The PPPHM [5] employs a public health framework to consider both the challenges and resilience experienced by children and families in pediatric healthcare settings. Based on the unique presentation of each pediatric patient and their family, evidence-based psychosocial care can be implemented along different tiers to match individual risks and strengths. The Universal tier represents the largest group of patients and families, those with understandable distress but also many strengths and abilities to successfully cope. At this level, interventions typically include psychoeducation, family-centered support, and ongoing screening for indicators of higher levels of risk. The Targeted tier includes a smaller group of patients and families with acute distress and identifiable risks and needs. Interventions are specific to these risks and needs, and distress and coping continue to be monitored. Patients and families presenting with persistent and/or escalating distress and high risk are served by interventions within the Clinical/Treatment tier, which often includes involvement of a behavioral health specialist (i.e., psychologist and/or psychiatrist) and more intense psychosocial services. Utilizing this type of framework allows for comprehensive and preventative care for all families, with allotment of specialized services to those most in need. Moreover, systematic screening and implementation of risk-based intervention allows for more equitable and bias-free provision of care [7].

In 2012, the Psychosocial Standards of Care Project for Childhood Cancer (PSCPCC) was formed to identify and address existing gaps in psychosocial care for children with cancer. Following extensive research and collaborative meetings, the PSCPCC published the Standards for Psychosocial Care of Children with Cancer and their Families in 2015 [6,8]. These 15 evidence-based standards outline the specific psychosocial needs of pediatric oncology patients and their families, and all pediatric oncology institutions and care centers are encouraged to follow them. The Standards can also serve as a guide for institutions seeking to expand their existing psychosocial programs or create new initiatives.

A key aspect of the Standards for Psychosocial Care is their emphasis on the importance of interdisciplinary collaboration [9]. While influenced by each cancer center’s size, patient volume, funding, and staffing, various clinical disciplines can contribute to implementing the Standards. In a survey of 144 pediatric oncology programs, the majority (over 93%) reported employing at least social workers and child life specialists to meet the psychosocial needs of patients and families [10]. Other psychosocial disciplines often represented in care teams include psychology, psychiatry, neuropsychology, spiritual care, creative arts, and school personnel [10,11]. With multiple disciplines contributing to the care of pediatric patients and their families, there are rich opportunities for interprofessional collaboration.

In addition to engaging multiple disciplines in the delivery of health care and development of new programs, inclusion of the patient-family voice is critical to the success of a health care system. A partnership between health care staff and families is mutually beneficial and underscores the importance that families play in the quality and safety of a health care system [12,13]. Moreover, a health care system’s commitment to patient-family-centered care (PFCC) reflects efforts to be continually responsive to the needs of each patient and family, recognizing that each family’s needs are different. Importantly, collaboration with patients and families is one of the core tenets of PFCC, reflecting the vital role of the family voice in a health care system, including its development, implementation, and evaluation of programs [14,15].

Using both The Standards and the PPPHM, the Psychosocial Services department at our institution collaborated with parent/caregiver advisors to develop and implement various programs to support caregivers and siblings of our patients. Psychosocial support programs were established across three tiers of support, based on the family member’s need. This paper outlines the development and implementation of these programs to provide a guiding reference for other institutions interested in establishing similar services.

## 2. Materials and Methods

Our children’s hospital serves children and young adults with cancer, blood disorders, and other catastrophic diseases. Patients are referred to our institution from all 50 states and internationally. In 2024, we served almost 9870 patients from over 64 countries: 62% of patients identified as White, 32% as Black or African American, and 6% identified as Other. Of the total patients served, 6241 (63%) were treated for a cancer diagnosis. Most pediatric patients at our institution are accompanied by at least one adult caregiver for all outpatient appointments and inpatient hospitalizations. Often, patients are accompanied by multiple caregivers (e.g., parents, grandparents, etc.) and child or adolescent siblings. These immediate and extended family members often benefit from psychosocial support and programming.

Two years following the publication of the Standards [6], our children’s hospital merged existing psychosocial disciplines into a unified department. These include child life, music therapy, psychology, school, social work, and spiritual care. Programs for teens and emerging adults, oncology transition, and staff support were later added. While each department and program has its own discipline leaders, they share a reporting structure directed by a psychosocial vice president, who is a pediatric psychologist. The department maintains close relationships with other services, such as psychiatry and palliative care. This restructure led to opportunities for aligned strategic planning, program development, clinical care delivery, and research and quality improvement efforts. To create caregiver and family supports, the newly formed department focused on clarifying scopes of practice of the different disciplines as well as the creation of a task force to specifically focus on identifying and meeting caregiver needs.

### 2.1. Development of a Scope of Practice Document to Enhance Collaboration

To enhance interprofessional collaboration and ensure observance of the Standards for Psychosocial Care, our Psychosocial Services department worked to clearly articulate the scope of practice for each psychosocial discipline at our institution [16]. In 2018, the directors of each psychosocial department convened multiple times alongside the department chief to discuss scopes of practice. Through this process, the group gained a better understanding of each discipline’s roles while identifying areas of overlap and potential programmatic gaps in care. Over a period of six months, staff meetings and feedback sessions provided opportunities for open discussion and consensus. Clinical disciplines also ranked their expertise and effort in each service area to reduce effort duplication. The resulting Scope of Practice Grid (SPG) is a “living” document that is reviewed and modified when needed to reflect new programs or services, evolving research, or changes in clinical practices.

Ultimately, the SPG enhances interprofessional collaboration by highlighting the strengths and areas of practice expertise for each psychosocial discipline at our institution. This shared understanding and clear role delineation also helps reduce any potential for professional “turf wars”. As we consider ways to enhance existing services or address gaps in psychosocial care, the identified areas of shared overlap help us determine which disciplines might be poised to implement new interventions. For example, our child life and social work departments recently partnered to launch a co-facilitated support group for siblings. Each clinician brings their discipline’s unique strengths and expertise into the group sessions, ultimately enriching the psychosocial support provided.

Areas of overlap are also addressed in a weekly psychosocial rounds meeting. Psychosocial rounds provide an opportunity to review the needs of patients and families and plan for co-treatment opportunities. Psychosocial Services clinicians can meet separately with patients and family members to provide clinical support, or they may collaborate, using their combined skill sets to enhance a family’s wellbeing. Psychosocial rounds are also a structured opportunity for staff to discuss any conflict or issues that arise due to role overlap and plan for how to best support the patient and family moving forward.

### 2.2. Development of Caregiver Support Task Force

A Caregiver Support Task Force was established to enhance the supports provided to caregivers to ensure alignment with Standard 6 (“Parents and caregivers of children with cancer should have early and ongoing assessment of their mental health needs. Access to appropriate interventions for parents and caregivers should be facilitated to optimize parent, child, and family well-being” [17]). Initially, the task force included one representative from each of the psychosocial services and three representatives from our PFCC program, including a caregiver advisor. As new programs were implemented and additional needs were identified, representation of the task force expanded to include additional staff from the Psychosocial Services department and the PFCC program, as well as members of Information Services, Patient Experience, and the palliative care service. Program development and implementation continued at a steady rate, and additional workgroups were established to capture the varying needs of different family members. While initial efforts focused exclusively on caregivers, programs were ultimately developed to address the needs of families more broadly, which dovetails nicely with Standard 4 (“All youth with cancer and their family members should have access to psychosocial support and interventions throughout the cancer trajectory and access to psychiatry as needed” [11]), Standard 7 (“Youth with cancer and their family members should be provided with psychoeducation, information, and anticipatory guidance related to disease, treatment, acute and long-term effects, hospitalization, procedures, and psychosocial adaptation. Guidance should be tailored to the specific needs and preferences of individual patients and families and be provided throughout the trajectory of cancer care” [18]), and Standard 10 (“Siblings of youth with cancer should be provided with appropriate supportive services. Parents and professionals should be advised about ways to anticipate and meet siblings’ needs, especially when siblings are unable to visit the hospital regularly” [19]).

## 3. Results

**Figure 1:**
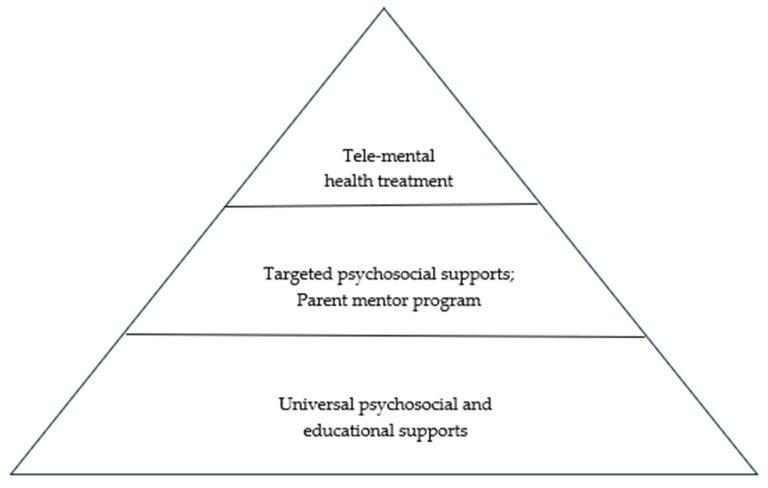
Three-tiered model of caregiver supports.

To effectively address the psychosocial standards of care, we followed the PPPHM three-tiered model of support [1] to develop a three-tiered model of caregiver support (see Figure 1) tailored to meet each family’s unique needs [20]. Recognizing that individuals and families have different preferences for receiving psychosocial support, we have been intentional to ensure programming opportunities are diverse. Some of the programs developed include psychoeducational materials and workshops, podcasts, support groups, animal-assisted therapeutic offerings, and individual mentoring or counseling. Clinicians across psychosocial services were instrumental in the development, implementation, and support of all programming, in collaboration with additional family-serving departments, such as Family Guest and Volunteer Services (see Table 1).

### 3.1. Universal Tier Programs

Caregiver Connections is a weekly support group for parents or other primary caregivers to find connection and meaning through shared experiences. The group aims to help decrease caregiver isolation by providing a space for caregivers to share about their child’s illness and treatment. Caregivers also receive psychoeducation and anticipatory guidance, including coping skills to manage common emotions like fear, stress, and/or fatigue. Licensed social workers facilitate the groups to help ensure a safe and supportive environment. When possible, a parent advisor attends, reinforcing our model of interprofessional program development and support. Since the inception in spring 2023, 109 sessions of Caregiver Connections have been scheduled. While some sessions have had limited or no attendance due to various challenges (e.g., patient/caregiver schedule conflicts, hesitation to attend a group support offering, patient treatment demands, etc.), there have been 107 attendees in total. A recent caregiver attendee encouraged other parents to participate in Caregiver Connections by posting a written testimonial to a social media page whose membership comprises caregivers of patients at our institution.

While Caregiver Connections focuses on adults, Sibling Connections is designed to provide the siblings of our patients an opportunity to make meaningful connections with others and receive psychosocial resources and education to support their unique needs. This programming is a collaboration between two disciplines, with groups co-facilitated by a child life specialist and a licensed social worker. Six sessions of Sibling Connections have been scheduled since its launch in summer 2024, with 12 children and adolescents participating thus far.

To protect the privacy of our caregivers and siblings who are not identified patients of our institution, we do not document sessions of either group in an electronic health record or collect specific identifying data. As a result, we do not have a measure of how often an individual caregiver or sibling returns to group as a measure of success. We have considered exploring methods of measuring progress in the future, but at this time, our primary focus has been on implementing the programs and making them available to our families.

The Caregiver Project is another collaborative initiative aimed at meeting the individualized needs of caregivers. Facilitated by child life and music therapy, caregivers record personal stories that honor and capture their experiences. This serves as a form of memory-making and legacy building for the caregivers of our patients. Beginning in May 2024, the Caregiver Project has been offered monthly for an hour and encourages caregivers to participate as their schedule allows.

The Paws at Play program was created to provide support to patients and families through one-on-one and group visits with one of three trained facility dogs. The dogs are each utilized for specific interventions and work alongside clinicians in the following departments: child life, psychology, social work, and the school program. While the Paws at Play program was created as a Universal tier support, the facility dogs can be utilized within Targeted and Clinical/Treatment tiers as well. When providing feedback about this program, one of our facility dog handlers noted that “[the dog] provided a calming presence for a patient whose anxiety impacted their treatment…”. Another handler recently shared the following about their patient: “A teenager recently received bad news about his illness. In the past, he never spoke to the doctor himself, and always asked his caregiver to do it for him. However, when the facility dog was there, he asked all his questions and was able to provide his input regarding his treatment preferences. The patient and his caregivers stated they felt it was because the facility dog was in the room providing him the comfort he needed to speak up”. A third handler shared, “the facility dog served as the conduit for me to provide anticipatory grief counseling and support to a family whose patient was at the end of life”.

To provide anticipatory guidance, we recently developed a psychoeducational toolkit and workshop to promote relationship health, resilience, and healthy communication. Content for the toolkit and workshop was developed by psychosocial providers utilizing interviews from couples with lived experience to create practical suggestions for supporting a partnership during a child’s treatment. We plan to develop additional psychoeducational toolkits and accompanying workshops on various psychosocial topics in the future. Additionally, members of the Psychosocial Services department have worked collaboratively to contribute articles to a freely available website, *Together by St. Jude™* [21], that provides anticipatory guidance and psychoeducation to patients and families within and outside of our institution. The site’s content is aimed at supporting patients and families impacted by childhood cancer, blood disorders, and other catastrophic illnesses and is translated into 12 languages.

Finally, the Caregivers SHARE podcast [22] was created to provide a place for family caregivers to hear advice, reflection, encouragement, and real-life stories from families and hospital care providers. As of January 2025, two seasons of the podcast have been created. Over the past year, episodes have been downloaded 1746 times in 21 countries around the world. One caregiver listener shared the following feedback and consented to have it included in this article: “We are inpatient this week, and I found myself needing something for encouragement at a time when we are struggling. So much of [the podcast] resonates with me, and I was impressed by the honest and hard, but hopeful way, in which the information was shared”.

### 3.2. Targeted Tier Programs

While all families can access programming from the Universal tier, families needing additional support can also access individual interventions with our psychosocial service clinicians as part of the Targeted tier. Because families often present with complex needs, interventions at the Targeted tier may also consist of co-treatment sessions between two or more psychosocial clinicians. Moreover, it is possible that individual sessions may build on information provided through programming at the Universal tier, in more depth.

A parent/caregiver mentor program was designed to meet the individualized needs of parents or adult family members of patients. Through the program, caregivers are matched with another caregiver whose child may have had a similar illness or treatment experience. Mentors provide insight into the treatment experience by drawing on skills or techniques they have learned to support coping. Both the mentor program and Caregiver Connections are scalable to serve a broad population. Patients and families at our institution have reported that one of their strongest supports is the friendship and connection with other families made through such programming or while at the hospital during treatment. A bereaved caregiver provided the following feedback about the mentor program and consented for it to be included in this article: “My mentor was a great asset to have during this difficult journey… We shared the stories of our children’s cancer journeys and shed a few tears. She gave me insight on what grief could look like in the future”.

### 3.3. Clinical/Treatment Tier Programs 

The Clinical/Treatment tier addresses the most significant level of individual need. Caregivers with preexisting mental health problems may require continued treatment so they can best care for their child [23]. Alternately, they may become aware of the need for mental health services due to their change in circumstances [24]. It is often difficult for caregivers to access services in an unfamiliar community, and childcare resources may be limited [25,26]. For these reasons, we have partnered with an outside agency to provide tele-mental health services, including psychotherapy and medication management, to parents or primary caregivers with significant needs. Caregivers are referred to the tele-mental health provider by our social work department and are eligible for ten sessions per calendar year while their child is on active treatment. They are also eligible for ten additional sessions if their child is enrolled in hospice and an additional ten sessions if they become a bereaved caregiver. Privacy concerns are limited, as minimal information is shared between the telehealth company and our institution, and all documentation related to services is performed within the tele-mental health company’s medical record.

This program began in late 2019. To date, a total of 488 referrals have been made to the tele-mental health program: 386 referrals were for therapy, 17 were for hospice caregivers, 81 for bereavement services, and 4 additional referrals were made without a specific designation. Not all caregivers who are referred to the program ultimately proceed with services; however, as of this date, the program has met the needs of 203 total families. This includes 155 caregivers whose children were receiving active treatment, 8 for hospice caregivers, 38 caregivers receiving bereavement services, and 1 referred without designation. Primary reasons caregivers did not receive services included caregiver refusal and inability of the tele-mental health company to reach them. Continuity of services has also been challenged by telehealth licensing laws, which require that clinicians be licensed in the state where a client is physically located [27]. Because our population is unique in that patients and families may reside anywhere in the United States or internationally, recipients of these services may have to shift providers when they return home from our institution. Fortunately, the tele-mental health agency we currently utilize has clinicians licensed in every US state, as well as those who are bilingual in Spanish and English. Because the agency is large, provider availability has not been a concern. While access to technology has not been an issue for most of our caregivers, we can provide a loaner device owned by the institution, if needed. In addition, flexible appointment times (e.g., evenings, weekends) and availability of hospital volunteers to watch the caregiver’s child allow for increased practicality of receiving services.

## 4. Discussion

After merging existing psychosocial services into a unified department, we have worked to steadily refine and improve the care we provide to pediatric oncology patients and their families. We utilized the Standards for Psychosocial Care of Children with Cancer and their Families [6,8] to identify outcomes, and our system was modeled after the three-tiered structure discussed in the PPPHM [5]. Within this, psychosocial supports for caregivers were created to provide a wide array of resources in various routes or formats (e.g., written material, podcasts, in-person interactions, and virtual visits; individual versus group formats), delivered by various individuals (e.g., peer versus psychosocial clinician), and with different timing (e.g., drop-in groups, scheduled groups/activities, individually scheduled visits, and on-demand content like podcasts and written material). This allows caregivers some degree of freedom to self-identify interventions that could work best for them, consistent with prior research demonstrating the importance of caregiver choice and recognition that individual family needs will vary [28,29]. It also allows for a more equitable approach to caregiver support and the ability to increase the level of services for those with higher needs, while providing targeted interventions to those with moderate needs and universal support to all caregivers. Many of the supports offered at the universal and targeted tiers are of low or no cost to our institution. They are implemented by our psychosocial clinicians using materials that are already available (i.e., space for group sessions) or present within their respective departments.

While we believe there are many strengths to the psychosocial support programs we have created for our caregivers, there are several limitations to our approach that we must acknowledge. First, our hospital serves primarily pediatric oncology patients. Given that we do not specifically recognize caregivers as patients, and to avoid encountering concerns related to dual roles, provision of the Clinical/Targeted tier of individualized mental health care for caregivers has been delivered through referral to tele-mental health providers. While this eliminates the potential for dual roles and may make it easier for those referred to feel comfortable that their own care will not influence their child’s care, it also may require a caregiver to take time away from their child to receive this needed treatment. While the use of telehealth limits this, not all caregivers are amenable to tele-mental health. We are aware that some other institutions will provide direct counseling and medication management to caregivers and identify them as patients of their facility as well. This approach has the benefit of ease of access and possibly greater familiarity of caregivers with the person and place delivering this care.

Another significant limitation to our work is that many hospitals and psychosocial service providers working within them will not have access to the resources to implement all of these supports, making exact replication of our model challenging. However, there are likely some supports that could be added to almost any other facility without a significant strain on limited resources. Our Caregivers SHARE podcast [22] and *Together by St. Jude™* website [21] are both freely available to the general public and are one way to provide or guide improvements to caregiver supports. In addition, printed education materials are an easy-to-develop and cost-effective intervention approach [30]. Lastly, development of a Scope of Practice document could allow psychosocial providers at other institutions to evaluate their current practices and identify areas of improved psychosocial supports to prioritize.

## 5. Conclusions and Future Directions

Overall, our work to develop and implement programs to better support families of youth with cancer provides the groundwork for future clinical and research endeavors. While we recognize not all of our interventions may be feasible or replicable at other institutions, we hope that our work can be a model for other institutions and psychosocial clinicians to develop and refine their own approaches to the psychosocial care of patients and their families alike. In the future, it will be important to perform prospective studies and to include objective measurement of program efficacy. As an example, our institution is currently piloting a caregiver distress screening tool that could provide insight into changes in caregiver distress associated with program usage.

## Figures and Tables

**Table 1:** Involvement of psychosocial services clinicians in the conceptualization, implementation, and support of new programming.

	Child Life	Music Therapy	Psychology	Resilience Center	School Program	Social Work	Spiritual Care
**Universal Tier**							
Caregiver Connections	S	S	S	S	S	C/I	S
Sibling Connections	C/I	S	S	S	S	C/I	S
The Caregiver Project	C/I	C/I	S	S	S	S	S
Psychoeducational Workshops	C/I	C/I	C/I	C/I	C/I	C/I	C/I
Podcast	C/I	C/I	C/I	S	S	C/I	C/I
Paws at Play	C/I	C/I	C/I	C/I	C/I	C/I	C/I
**Targeted Tier**							
Individual Interventions	C/I	C/I	C/I	C/I	C/I	C/I	C/I
The Mentor Program	S	S	S	S	S	S	S
**Clinical Tier**							
Tele-Mental Health Program	S	S	C/I	S	S	C/I	S

Note: Conceptualization (C), Implementation (I), and Support (S).

## Data Availability

Data available upon request.
